# Modern Synthetic Strategies with Organoselenium Reagents: A Focus on Vinyl Selenones

**DOI:** 10.3390/molecules26113148

**Published:** 2021-05-25

**Authors:** Martina Palomba, Italo Franco Coelho Dias, Ornelio Rosati, Francesca Marini

**Affiliations:** Group of Catalysis, Synthesis and Organic Green Chemistry, Department of Pharmaceutical Sciences, University of Perugia, Via del Liceo, 06123 Perugia, Italy; martina.palomba@unipg.it (M.P.); italo.francocoelhodias@studenti.unipg.it (I.F.C.D.); orneli.rosati@unipg.it (O.R.)

**Keywords:** selenium, domino reactions, heterocycles, natural products, spiro compounds, annulations, enantioselective synthesis, organocatalysis

## Abstract

In recent years, vinyl selenones were rediscovered as useful building blocks for new synthetic transformations. This review will highlight these advances in the field of multiple-bond-forming reactions, one-pot synthesis of carbo- and heterocycles, enantioselective construction of densely functionalized molecules, and total synthesis of natural products.

## 1. Introduction

**Figure 1:**
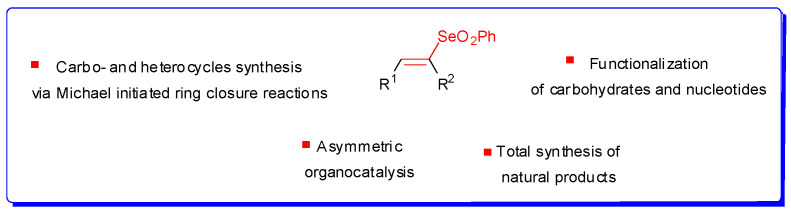
Vinyl selenones as versatile reagents in organic synthesis.

Selenium chemistry has rapidly grown over the past years and nowadays selenium reagents are recognized tools in the chemo-, regio-, and stereoselective synthesis of molecules containing selenium or not [1,2,3]. In fact, selenium functionalities can be easily reduced, eliminated or transformed into other functional groups with high stereocontrol. In this field, over the past decade, the chemistry of vinyl selenones has been the object of renewed interest, leading to the development of diverse and powerful methods with applications in challenging fields of organic synthesis, such as the enantioselective synthesis and the total synthesis of natural products. The chemistry of these hexavalent, tetra-coordinated selenium compounds shows interesting analogies with that of the corresponding vinyl sulfones. Both contain an electron-withdrawing group that stabilizes vicinal carbanions and activates the double bond to conjugate nucleophilic attack, but the weak C-Se bond donates to the phenylselenonyl moiety, a better leaving group character for further substitution or elimination reactions. In this review, recent applications of vinyl selenones in domino or sequential one-pot processes for the assembly of carbo- and heterocycles, the functionalization of biomolecules, and the organocatalyzed enantioselective construction of all-carbon quaternary stereocenters will be presented and discussed (Figure 1). Main advantages of such multiple-bond forming strategies are the mild and operationally simple reaction conditions, the good to excellent yields, and the high chemo-, regio-, and stereo-selectivities. Examples of use of vinyl selenones in the total synthesis of natural products will also be shown.

## 2. Synthesis of Vinyl Selenones and Their Biological Activities

**Scheme 1:**
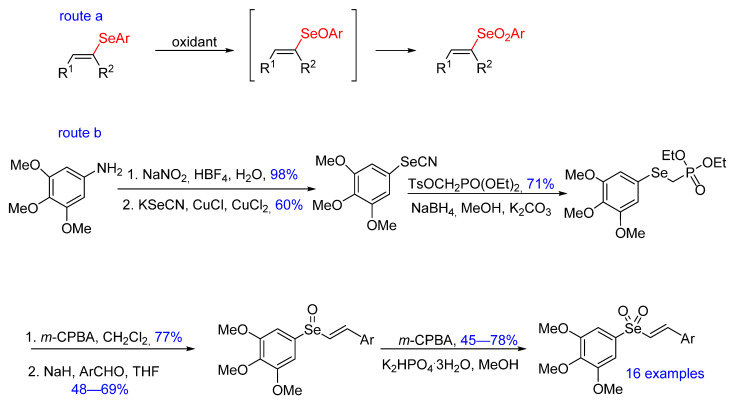
Synthetic approaches to vinyl selenones.

Selenones are usually prepared by oxidation of the corresponding selenides [4,5]. The first oxidation of selenides to selenoxides is fast, but the decreased electron density on the selenium atom makes selenoxides less prone to the second oxygen transfer. Even if, several strong oxidants convert selenides into selenones, i.e., KMnO_4_ [6], peroxyacids or their salts [6,7], H_2_O_2_ in the presence of benzenseleninic acid [8], HMPA peroxo complex of molybdenum [9], Oxone^®^ [10], and HOF·CH_3_CN complex [11], 3-chloroperoxybenzoic acid (*m*-CPBA) [12] is the reagent of choice for the oxidation of vinyl selenides (Scheme 1, route a). It is used in excess in alcoholic, ethereal or halogenated solvents. Recently, the greener Oxone^®^ was used to generate vinyl selenones [13] in water without addition of any catalyst or co-solvent.

**Figure 2:**
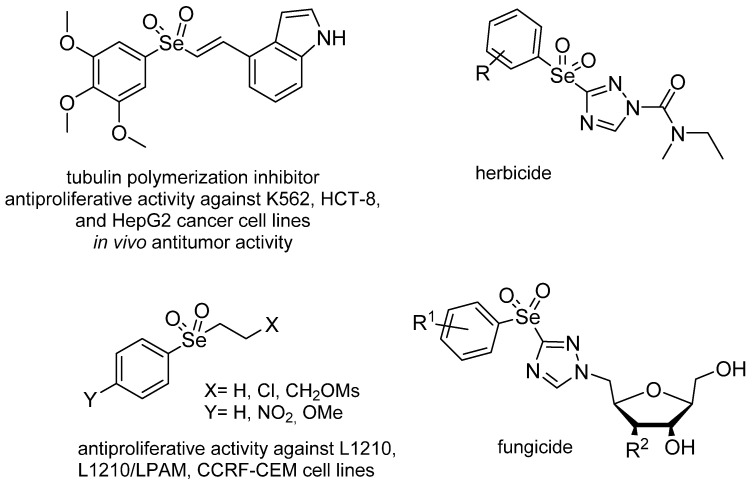
Biologically active selenones.

In recent years, selenium-containing compounds have showed interesting biological activities and a great potential in medicinal chemistry, particularly in cancer therapy [14]. Bioactive vinyl selenones were recently prepared through the multistep sequence described in Scheme 1, route b. In this protocol the Wittig–Horner reaction between phenylseleninylmethyl phosphonates and variously substituted aromatic aldehydes generated vinyl selenoxides which were transformed into the corresponding selenone by oxidation with *m*-CPBA [15]. These compounds were evaluated for their in vitro and in vivo anticancer activities. They showed tubulin polymerization inhibition and antiproliferative activity against several cancer cell lines. The structure of the most active compound is reported in Figure 2 together with other diaryl or aryl alkylselenones with antiproliferative [16] or pesticide activities. [17,18]. 

## 3. Michael-Initiated Ring Closures

**Scheme 2:**
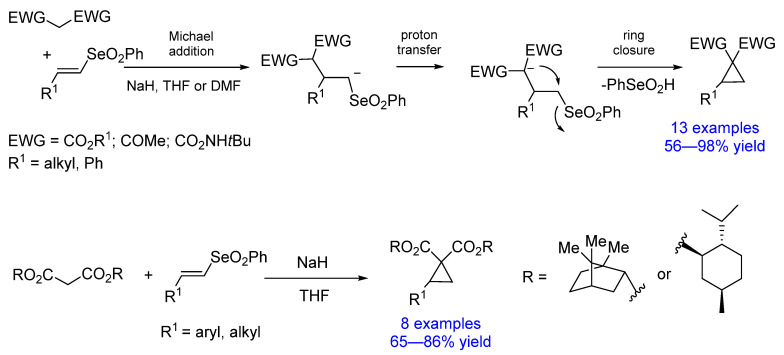
Cyclopropanations of active methylene compounds with vinyl selenones.

Michael initiated ring closure reactions of vinyl selenones demonstrated to be highly efficient in the regio- and stereoselective synthesis of small cycles. Cyclopropanes have attracted great attention by the scientific community. These small rings are present as key structural motif in several natural products and drugs and are used as synthetic intermediates due to the easy ring opening. Since the seminal independent works by Kuwajima and Tiecco [19,20,21], vinyl selenones and several active methylene pronucleophiles, such as dialkyl malonates, β-ketoesters, and β-ketoamides, have been employed for the construction of these small rings. Nitromethane was also a competent pronucleophile. The reactions proceed in the presence of strong bases through a deprotonation/Michael addition, followed by a proton transfer and a ring closure reaction by nucleophilic displacement of the selenium moiety. Thus, the electron-withdrawing benzenselenonyl group, first activates the alkene to the Michael addition and then promotes the cyclization acting as an excellent leaving group (Scheme 2). Benzeneseleninic acid is generated as the by-product.

**Scheme 3:**
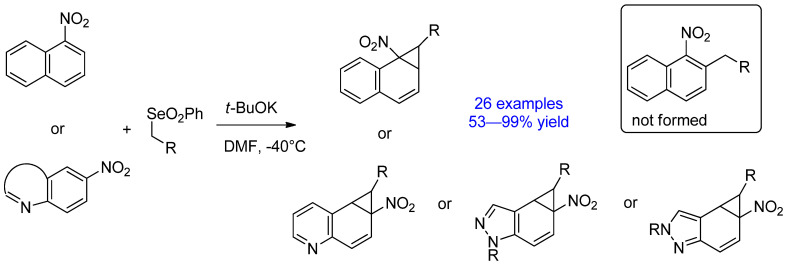
Alkyl selenone-mediated cyclopropanations by dearomatization of nitronaphthalenes and derivatives.

In 2009, this strategy was applied to the asymmetric synthesis of cyclopropanes starting from malonates containing (−)-bornyl or (−)-mentyl groups as chiral auxiliaries [22]. These reactions occurred with low diasteroselectivity, but the mixtures of the two diastereoisomers were easily separated by chromatography, giving access to enantiomerically pure compounds. Some products were successfully converted into α-cyclopropane-α-amino acids (ACCs) in two steps. Enantiopure ACCs find interesting applications in pharmacology and bioorganic chemistry. In fact, they are conformationally constrained analogues of proteinogenic aminoacids and play an important role in the synthesis of peptidomimetics and foldamers. Very recently, alkyl phenyl selenones were involved in an interesting cyclopropanation reaction by dearomatization of 1-nitronaphthalenes [23]. Contrary to the previously mentioned processes, this domino reaction, employs alkyl selenones as Michael donors in a Corey–Chaykovsky type cyclopropanation (Scheme 3). The experiments demonstrated that sterically demanding substituents at the selenium nucleophile favor the formation of cyclopropanes and suppress the competitive formation of the alkylated products by a Michael/elimination sequence. The reaction has been successfully extended to 2-nitronaphtalenes, 6-nitroquinoline, and isomeric 5-nitroindazolines. Mixtures of *endo* and *exo* isomers were usually isolated.

**Scheme 4:**
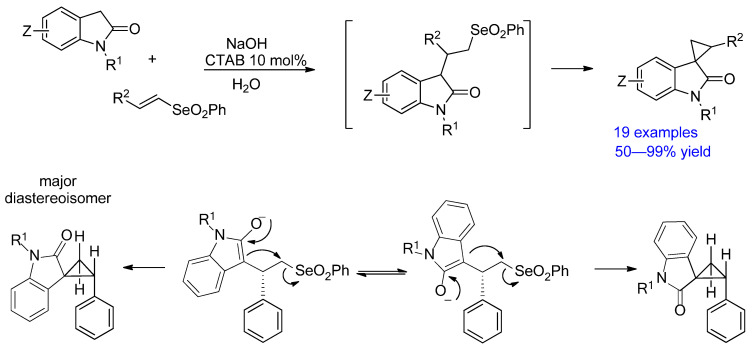
Spirocyclopropyl oxindoles via a Michael addition/cyclization cascade.

In the past decade, the development of new methods for the synthesis of spirocycles became a hot topic in organic synthesis. The conformational rigidity, the three-dimensional nature with substituents in well-defined spatial disposition, the improved physicochemical and pharmacokinetic properties and the relative structural novelty make spirocycles attractive leads in drug discovery programs [24,25]. In fact, compared to flat aromatic compounds, they seem to have more chances to maximize specific interactions with biomolecules. A number of new protocols have been described as a mean for generating structural diversity and/or impart efficiency and stereoselectivity to the cyclization. In this field, Marini and co-workers used 2-oxindoles as pro-nucleophiles for a facile assembly of spirocyclopropyloxindoles [26]. The synthesis of spirocyclopropyl oxindoles has been developed in aqueous basic conditions in the presence of catalytic cetyltrimethylammonium bromide (CTAB). As reported in Scheme 4, variously substituted spirocyclopropyl oxindoles with three point of diversity were isolated in good to excellent yields. High diastereoselectivities were observed starting from the β-aryl substituted vinyl selenones, probably due to the stabilizing π-π interactions between the oxindole ring and the neighboring aromatic ring in the transition state. Some selected spiro-compounds demonstrated anti-HIV-1 activity.

**Scheme 5:**
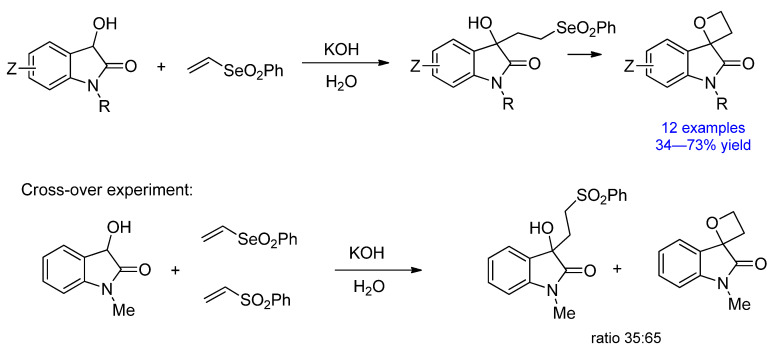
Synthesis of spirooxindole oxetanes: cross-over experiment between vinyl selenone and sulfone.

Aqueous basic conditions were also successfully employed for the construction of a challenging spiro 2,2-substituted oxetane motif in moderate to good yields [27]. The Michael addition/intramolecular etherification cascade was performed with N-protected or unprotected 3-hydroxy isatins and the phenyl vinyl selenone without addition of surfactants (Scheme 5). This reaction is a valid alternative to photochemical [2+2] cycloadditions (Paternò–Buchi reactions) or multistep protocols involving intramolecular Williamson etherifications in the preparation of spirooxindole oxetane. Limitations of these approaches are the narrow substrate scope and the poor yields, respectively. Authors also performed comparative studies among the vinyl selenone and its more common sulfur analogue, vinyl sulfone. Equal amounts of the two chalcogenones were allowed to react with a limiting amount of the 3-hydroxy isatin. Vinyl sulfone showed a poorer reactivity as Michael acceptor and a complete inability to cyclize under the standard reaction conditions.

**Scheme 6:**
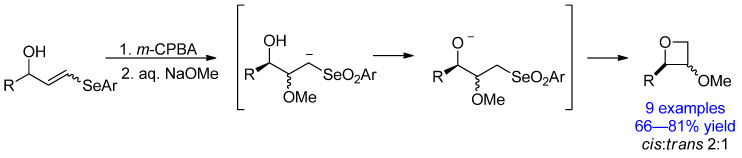
Oxidation/oxa-Michael/etherification cascade of 1-alkyl-3-phenylseleno-2-propen-1-ols.

A simple synthesis of methoxyoxetanes has been previously described by Kuwajima [28]. In this telescopic protocol, the 1-alkyl-3-phenylseleno-2-propen-1-ols were oxidized with *m*-CPBA and then treated with NaOH in MeOH. The methoxide ion adds to vinyl selenones generating oxa-Michael adducts that after proton transfer and intramolecular nucleophilic substitution gave methoxyoxetanes in good yields, as 2:1 mixtures of unseparable *cis* and *trans* diastereoisomers, independently of the geometry of the starting selenide (Scheme 6).

**Scheme 7:**
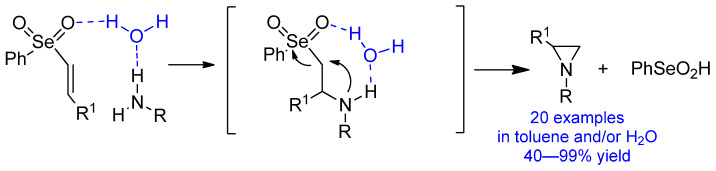
Aziridines by aza-Michael Initiated Ring Closure (aza-MIRC) of β-substituted vinyl selenones.

In 2010, a one-pot synthesis of aziridines from vinyl selenones and primary amines, aminoalcohols or diamines was described [29]. The aza-Michael Initiated Ring Closure reaction (aza-MIRC) was performed both in toluene or in aqueous suspension or emulsion, without addition of any catalyst or additive in good yields. The main limitation of this protocol was the lack of reactivity of aromatic amines. In this case, water also played a beneficial role, since higher reaction rates were observed in aqueous media. It seems reasonable that water may be responsible for a faster proton transfer. Moreover, the H-bonding network can facilitate either the conjugate addition or the cyclization step (Scheme 7).

**Scheme 8:**
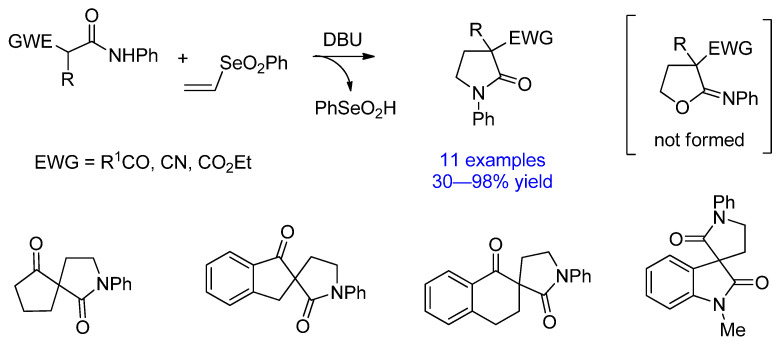
Synthesis of γ-lactams.

The one-pot synthesis of α,α-disubstituted γ-lactams from vinyl selenones and N-phenyl substituted amides has been reported (Scheme 8). Lactams were obtained in good to excellent yields via a Michael addition/intramolecular N-alkylation cascade [30].

The choice of the proper base was crucial for the success of the process. In fact, due to the bidentate nature of the nucleophilic amide, the formation of C-N or C-O bonds gave access to lactams or imidates, respectively. Both Et_3_N and K_2_CO_3_ gave consistent amount of the imidates, whereas 2 equivalents of the stronger base, 1,8-diazabicyclo[5.4.0]undec-7-ene (DBU), afforded lactams in high yields as the sole reaction products. This approach was extended to the synthesis of spirolactams containing a cyclopentanone, indanone, tetralone, or oxindole scaffold.

**Scheme 9:**
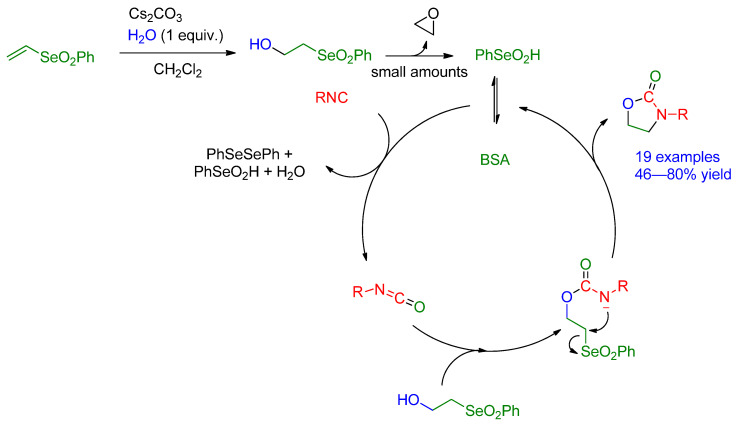
One-pot synthesis of oxazolidin-2-ones.

In 2016, Zhu reported that alkyl isocyanides react with phenyl vinyl selenone in the presence of 1 equivalent of water and a catalytic amount of Cs_2_CO_3_ affording oxazolidin-2-ones in good yields [31]. In this reaction four new bonds were generated and the phenyl selenenonyl group acted not only as an activating and a leaving group, but also as a latent oxidant. The plausible mechanism, reported in Scheme 9, was supported by control experiments carried out with *n*BuN^13^C and H_2_^18^O. Water adds to phenyl vinyl selenone under basic conditions to generate an oxa-Michael adduct that in small amounts cyclizes to give an oxirane (observed by ^13^C-NMR experiment) and benzenseleninic acid. The latter compound, in equilibrium with its anhydride (BSA), is responsible of the oxidation of the isocyanide to isocyanate. The nucleophilic addition of the Michael adduct onto the isocyanate generates a carbamate which can cyclize to oxazolidinone by the extrusion of benzenseleninic acid.

**Scheme 10:**
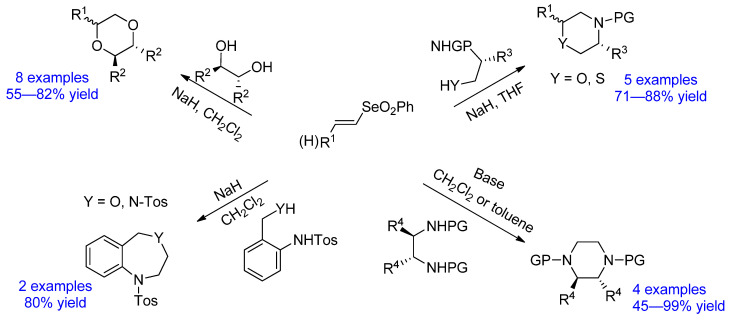
Synthesis of oxygen, nitrogen and/or sulfur-containing 6- and 7-membered heterocycles.

Interesting domino processes for the access to six or seven-membered heterocycles were developed by Bagnoli and co-workers in 2011 [32]. 1,4-Dioxanes, oxazines, thiazines and piperazines were synthesized through the formation of two new carbon-heteroatom bonds using vinyl selenones and commercially available chiral 1,2-diols, *N*-protected amino alcohols, thiols or diamines without loss of enantiomeric purity. The access to enantiopure piperazines is particularly interesting. This privileged scaffold is present in several pharmacologically active compounds, but there is a paucity of reliable and stereoselective approaches to carbon-substituted piperazines since most of the methods give access only to compounds with substituents at the nitrogen atoms. Moreover, 1,4-Benzoxazepines, and 1,4-benzodiazepines were prepared (Scheme 10).

**Scheme 11:**
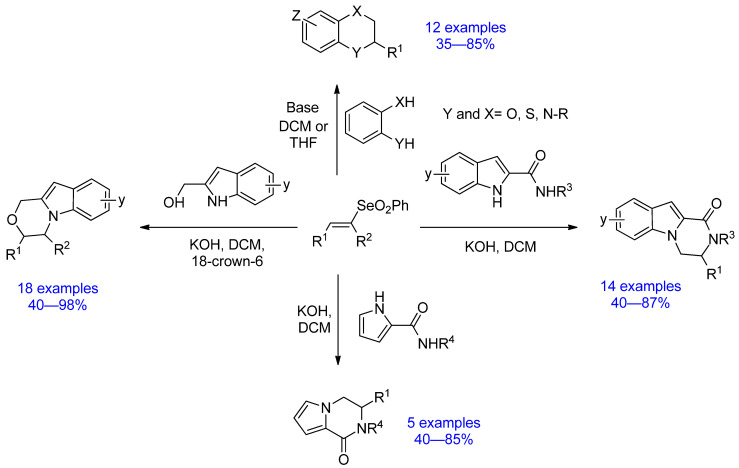
Synthesis of oxazino[4,3-a]indoles and pyrazino[1,2-a]indoles.

Scheme 11 shows similar one-pot conjugate addition/cyclization strategies for the synthesis of fused benzenes [33], indoles and pyrroles [34,35]. Such condensed heterocycles are very common in drugs and natural products. Carboxamides derived from enantiopure amino esters or amines (*ee* > 98%) also gave chiral pyrazino fused indoles and pyrroles in good yields. The addition of the complexing agent 18-crown-6 was essential to obtain products with high chemo- and regio-selectivity in the synthesis of oxazino[4,3-*a*]indoles.

**Scheme 12:**
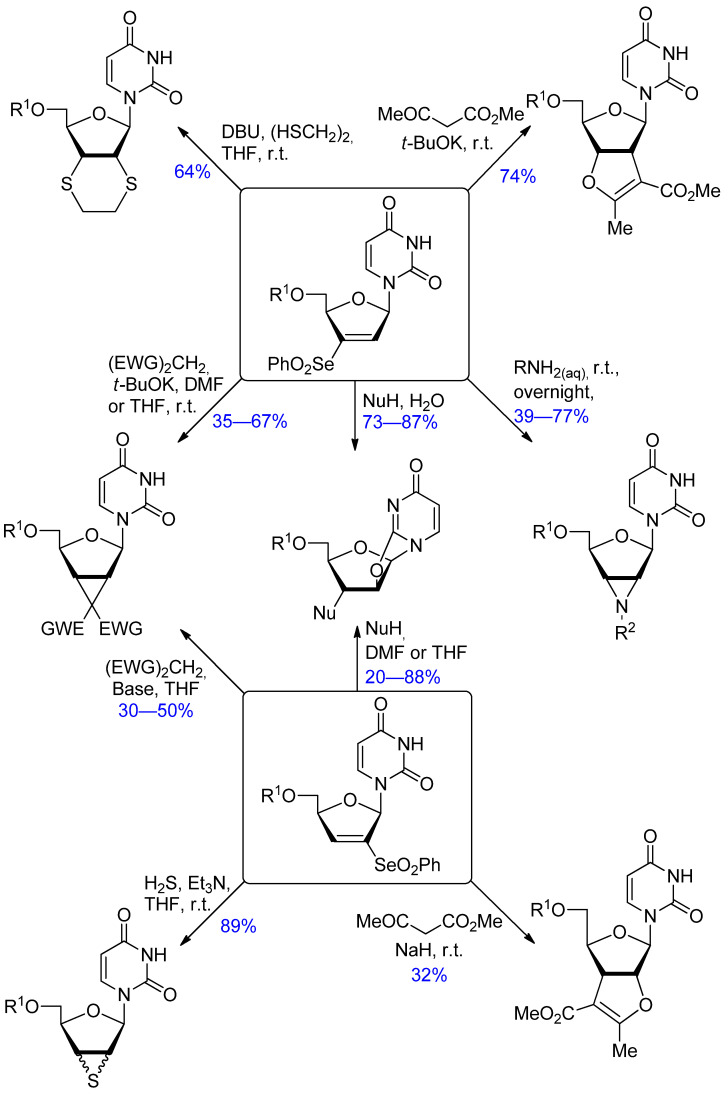
Uridine analogues synthesized through selenone-based transformations.

Vinyl selenone-modified nucleosides or monosaccharides were deeply investigated as substrates for the diversity-oriented synthesis (DOS) of enantiopure, structurally and functionally complex molecular scaffolds. In early reports, Chattopadhyaya et al. [36,37,38,39] described the synthesis of 2′,3′-modified uridine derivatives as part of a drug discovery program concerning new types of selective inhibitors of the HIV-reverse-transcriptase (Scheme 12).

**Scheme 13:**
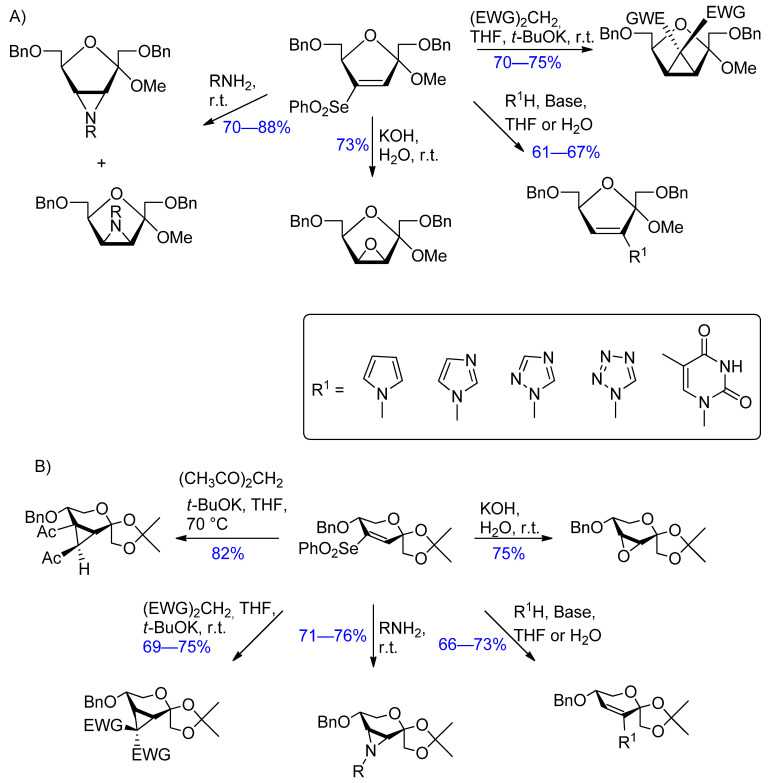
Vinyl selenone-based functionalizations of carbohydrates.

Once again, the formation of the products can be explained by the bis electrophilic properties of the 2′,3′-ene-2′-phenyl selenone which undergoes sequential Michael addition and nucleophilic substitution of the selenonyl group. The C2=O group of the uracil is sometimes involved as the internal nucleophile. The combination of the chirality of carbohydrates with the high reactivity of vinyl selenones can offer interesting opportunities for generating new stereochemically defined and structurally complex compounds. Recently, Pathak designed *D*-xylose or *D*-fructose-derived alkenyl selenones to explore Michael-initiated functionalizations of sugars [40,41] with small carbo and heterocyclic rings. Moreover, furanoside and pyranoside rings were decorated with five-membered N-heteroaromatic compounds through Michael addition/elimination sequences. Some examples with *D*-fructose-derivatives are collected in Scheme 13.

These reactions nicely complement vinyl sulfone-mediated transformations of sugars. In fact, the easy removal of the selenonyl moiety gave access to completely different reaction pathways [42,43].

**Scheme 14:**
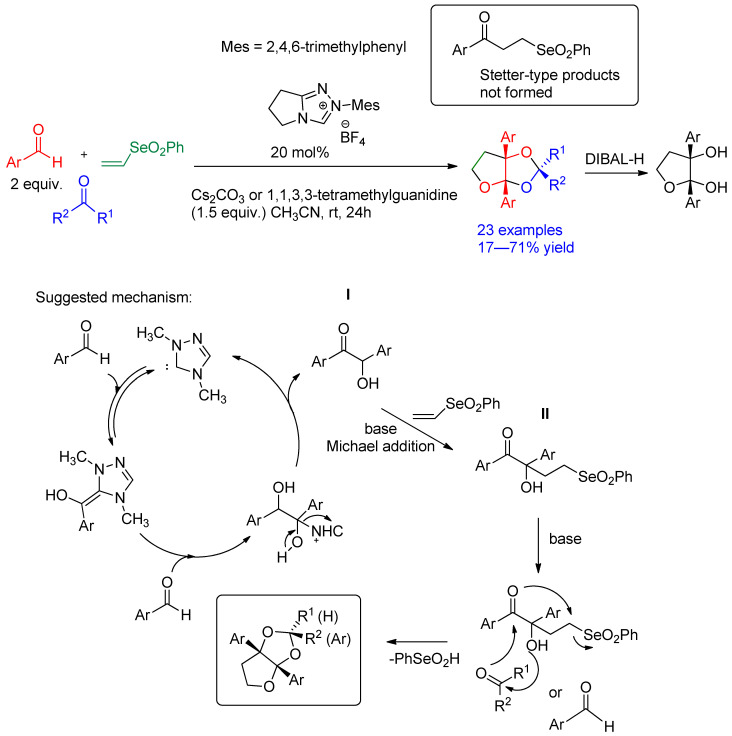
Functionalized 2,3-diaryltetrahydrofurans by NHC/base mediated multicomponent reactions.

In 2017, Tiwari described the *N*-heterocyclic carbene (NHC)/base catalyzed addition of aromatic aldehydes and ketones to vinyl selenones for the formation of multifunctionalized tetrahydrofuranes [44]. This unprecedented three-component reaction gave access to challenging 2,3-dihydroxy-2,3-diaryltetrahydrofurans with two contiguous oxygenated quaternary stereocenters (Scheme 14).

The following reaction steps can explain the formation of the products: (a) the NHC-catalyzed synthesis of the benzoin **I** from two molecules of aromatic aldehyde, (b) the base promoted Michael addition of the benzoin to the vinyl selenone to generate the intermediate **II**, (c) the reaction of **II** with acetone or 2,2,2-trifluoro acetophenone to generate tetrahydrofuro[2,3-*d*][1,3]dioxoles. A third molecule of aldehyde can also react with **II** in place of a ketone. Once again, results highlight the different reactivity of the vinyl selenones in respect to vinyl sulfones and vinyl phosphonates, which simply generate Stetter-type products under similar conditions. The bicyclic products gave the corresponding 2,3-dihydroxytetrahydrofuranes by treatment with DIBAL-H at room temperature for 24 h.

**Scheme 15:**
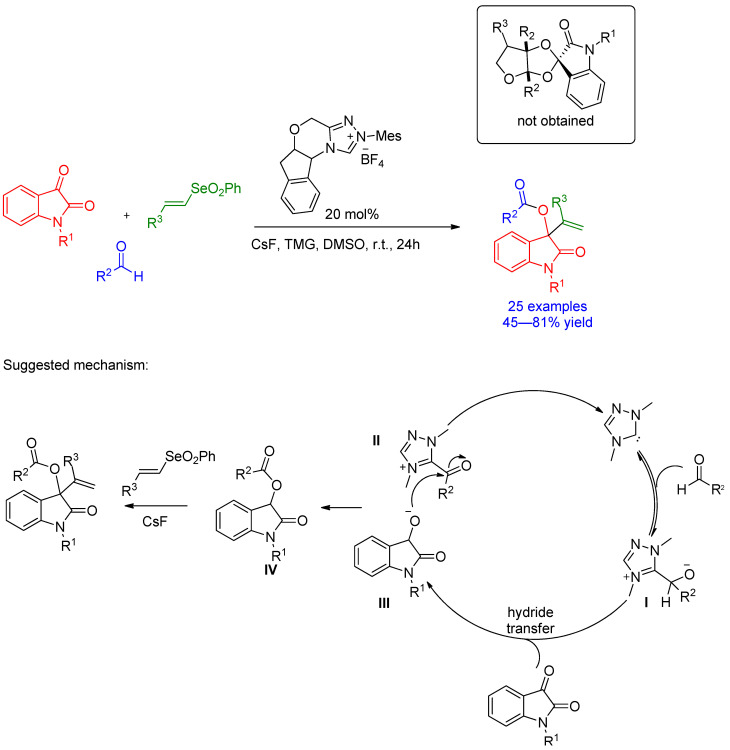
Synthesis of 1-substituted 3-vinyl oxindoles.

Interestingly, little changing in the substrate can completely alter the reactivity. For instance, isatins in place of the above mentioned ketones gave not access to the expected spiro compounds. Alkenylation products were obtained as a consequence of the NHC-catalyzed redox reaction described in Scheme 15 [45]. The intermediate **I**, generated when the aldehyde reacts with the *N*-carbene catalyst, undergoes a hydride transfer to generate the intermediate **II** and the anion **III**. The reaction between intermediates **II** and **III** affords a 2-benzoyl isatin **IV** that reacts with vinyl selenone via a CsF-assisted Michael addition. The following elimination of benzenseleninic acid gives variously substituted vinyl oxindoles in moderate to good yields.

## 4. Enantioselective Organocatalytic Transformations

**Scheme 16:**
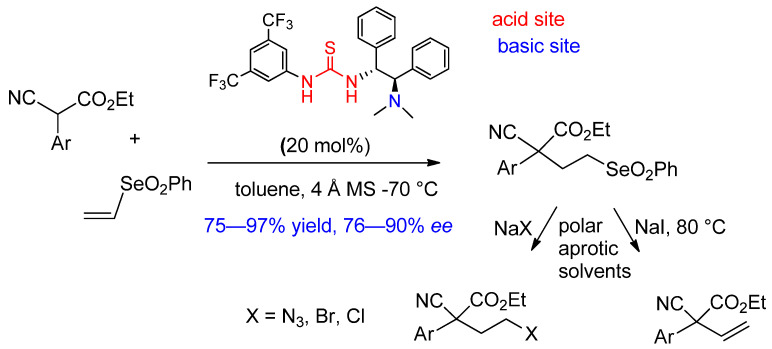
Enantioselective addition of 2-aryl-2-cyanoacetates to the vinyl phenyl selenone and chemical transformations.

Enantioselective organocatalysis consists in the conversion of prochiral or racemic substrates into highly enantioenriched products by effect of a chiral organocatalyst. In the last years this approach acquired great popularity as a robust and powerful strategy for the synthesis of chiral building blocks, natural products, and pharmaceutically relevant molecules as single enantiomers [46]. Common advantages of organocatalysts are: easy handling, low cost, poor toxicity, air and water stability. Moreover, the metal-free conditions are particularly useful in the production of pharmaceutical intermediates. In this field, privileged chiral organocatalysts with non-covalent activation mode, such as ureas, thioureas, C6′-hydroxyl cinchona derivatives, or squaramides have been conveniently employed in Michael-type reactions between vinyl selenones and different pro-nucleophiles constructing all-carbon quaternary stereocenters and/or multiple stereocenters with excellent levels of enantioselectivity. These organocatalysts are bifunctional small molecules bearing a hydrogen bond donor group (in red in the following schemes) besides a basic site (in blue in the following schemes) on a chiral scaffold. Usually, a tertiary amine at the basic site generates the nucleophile by deprotonation, while a weak Brønsted acid group activates the electrophile through hydrogen bonding. Thus, the catalyst simultaneously orients and activates both the Michael donor and the acceptor allowing that the addition occurs with an excellent level of stereocontrol. The first organocatalyzed conjugate addition of vinyl selenones was reported by Marini et al. in 2009 [47]. The reaction was an α-functionalization of 2-aryl-2-cyanoacetates (Scheme 16). After a careful screening, it was clear that the reaction is best carried out in the nonpolar aprotic solvent toluene and in the presence of 4Å molecular sieves. Products containing all-carbon quaternary stereocenter were obtained at −70 °C with a thioureidic catalyst in good to excellent yields and high enantiomeric excesses. A lower yield (53%) and a poorer enantioselectivity (70%) was observed with 2-allyl-2-cyanoacetate. The Michael adducts were transformed into synthetically valuable chiral intermediates, not directly accessible via conjugate addition, without loss of enantiomeric excess. The formation of the all-carbon quaternary stereocenter bearing a terminal double bond is particularly interesting. In fact, the α-vinylation of active methylene compounds is still in great demand, due to the lack of methods for the enantioselective installation of alkenyl groups at sterically hindered positions.

**Scheme 17:**
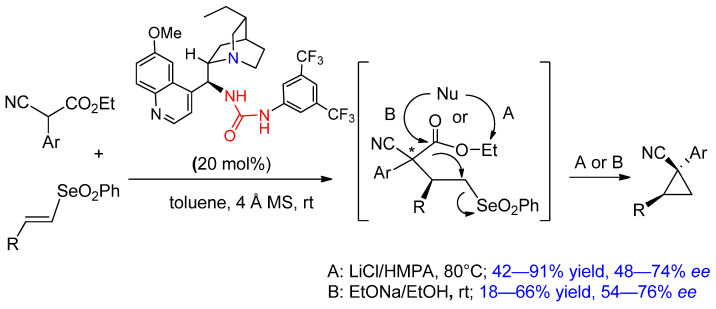
One-pot organocatalyzed enantioselective conjugate addition/cyclopropanation cascade.

Reactions carried out with the less reactive β-substituted vinyl selenones at room temperature were used for the synthesis of enantioenriched cyclopropanes with vicinal tertiary and quaternary stereocenters [48]. The sequential one-pot strategy is based on the nucleofugacity of the selenonyl group and the ability of the same group to control the regio- and stereoselectivity of the conjugate addition. Enantioenriched Michael adducts were generated under control of a bifunctional ureidic catalyst and cyclized by intramolecular nucleophilic displacement of the phenylselenonyl group induced by a de-ethoxycarbonylation process. Two different conditions were used for the de-ethoxycarbonylation step: a Krapcho-type protocol with LiCl in HMPA or the more eco-friendly treatment with EtONa in EtOH (Scheme 17). *Z*-Cyclopropanes were recovered as single isomers in moderate to high yields and acceptable to good enantiomeric excesses.

**Scheme 18:**
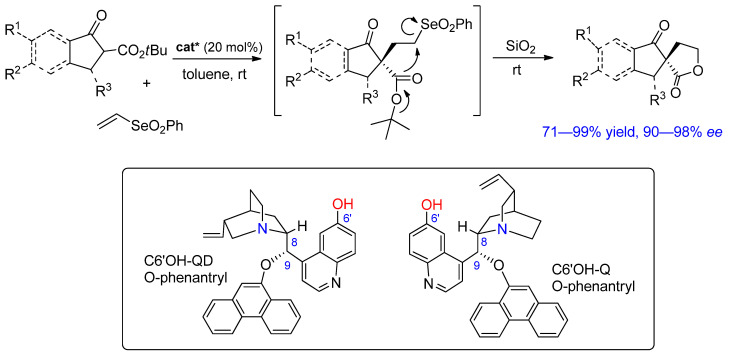
One-pot organocatalyzed enantioselective Michael addition/cyclization cascade for the synthesis of spirolactones.

Organocatalyzed Michael-initiated cyclizations for the enantioselective synthesis of polycyclic compounds, such as spirolactones [49] and β-aminoesters [50] with a tetrahydroindeno[1,2-*b*]pyrrole core, were also developed by the same authors. In these reactions, the selenonyl group also acts as a traceless agent able to activate and control the addition step. The key step of both synthetic protocols is the conjugate addition of a cyclic *tert*-butyl-β-ketoester to vinyl phenyl selenone, catalyzed by a O-9-phenantryl C6′-OH quinidine or quinine-derivative, that generates highly enantioenriched Michael adducts. In the first process, the addition of silica gel after the Michael reaction, promoted the ring closure through nucleophilic displacement of the SeO_2_Ph by the carbonyl ester. The presence of the *tert*-butyl residue, which can be easily removed by the free silanol groups of the silica, was essential for an efficient cyclization. The use of pseudo-enantiomeric catalysts O-phenantryl C6′OH-QD and C6′OH-Q, having opposite configurations at C8 and C9, provided spirolactones with comparable yields and enantiomeric excesses, but opposite enantioselectivity (Scheme 18).

**Scheme 19:**
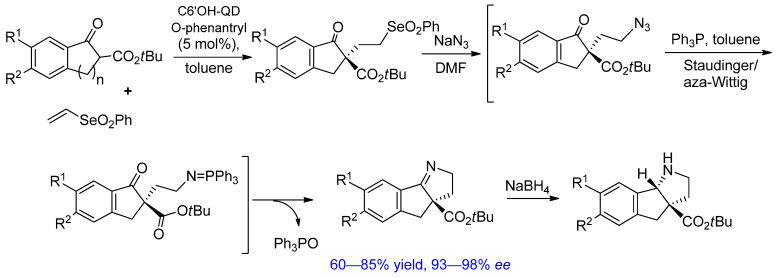
Telescopic organocatalyzed synthesis of enantioenriched dihydroindeno[1,2-b]pyrroles.

Alternatively, the Michael adducts were transformed into alkyl azides by an intermolecular nucleophilic substitution with sodium azide. The following Staudinger/intramolecular aza-Wittig sequence gave access to dihydroindeno[1,2-*b*]pyrroles. The three steps were carried out telescopically, with the exclusion of purification steps. Products were isolated by column chromatography in 60–85% yield and 93–98% enantiomeric excess. Cyclic β-aminoesters were obtained by reduction with sodium borohydride in MeOH (Scheme 19).

**Scheme 20:**
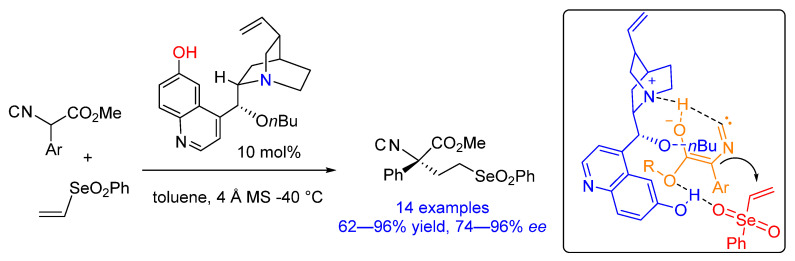
Enantioselective addition of 2-aryl-2-isocyanoacetates to the vinyl phenyl selenone.

Interestingly, a catalyst loading of only 5 mol% did not compromise the chemical and optical yields, albeit longer reaction times were needed. In more recent papers, the trifluoroacetic acid deprotection of the racemic β-amino esters obtained by a modified protocol that employs Na_2_CO_3_ in place of the chiral catalyst, gave access to β-amino acids. These compounds were resolved by amylase or zwitterionic cinchona alkaloid-based chiral stationary phases [51,52]. In 2013 Zhu and coworkers developed the asymmetric conjugate addition of 2-aryl-2-isocyanoacetates to vinyl phenyl selenone with the C6′-OH quinine-derived catalyst (C6’OH-Q-O*n*Bu), bearing a *n*-butoxy group reported in Scheme 20 [53]. In the same scheme, a possible transition state consistent with the observed enantioselectivity was also reported. The presence of electron-donating or electron-withdrawing groups at ortho, meta, or para position of the α-aryl substituent as well as the presence of heteroaromatic rings were well tolerated. In all the cases, the products were obtained in good to excellent yields and enantiomeric excesses ≥74%.

**Scheme 21:**
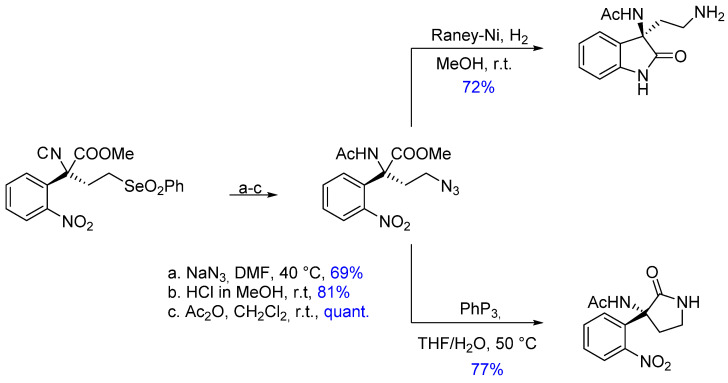
Synthesis of chiral 3,3-disubstituted oxindole and pyrrolidinone.

This method offers a facile access to versatile building blocks in a high enantioenriched form. Scheme 21 shows their easy transformation into pharmaceutically relevant heterocycles.

**Scheme 22:**
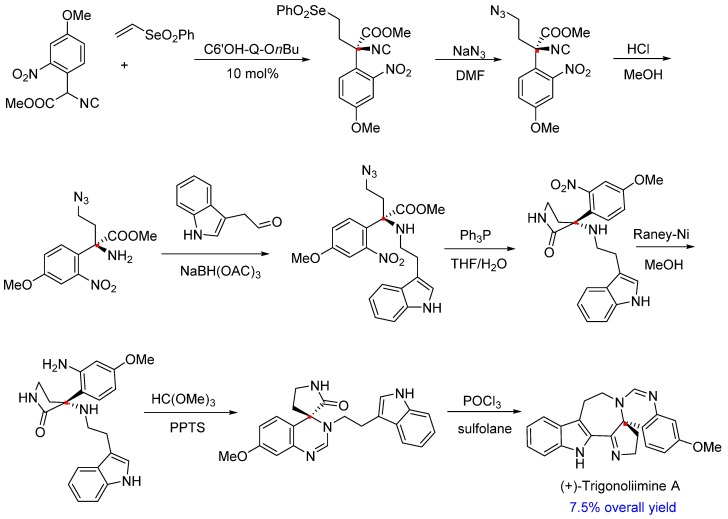
Total synthesis of (+)-trigonoliimine A.

The new methodology also found nice applications in the total synthesis of natural products. The first example is referred to the enantioselective synthesis of (+)-Trigonoliimine A (Scheme 22).

This alkaloid, with an unusual hexacyclic skeleton and a quaternary carbon center, was isolated from the extract of the leaves of *Trigonostemon Li.* It is known for the modest anti-HIV activity. After the Michael addition and the substitution of the phenylselenonyl group with sodium azide, the synthetic sequence proceeds with the hydrolysis of the isonitrile. The next step was the reductive amination with 2-(1H-indole-3-yl)acetaldehyde in the presence of NaBH(OAc)_3_. Then, a Staudinger reduction was performed to directly produce a lactam. Finally, the reduction of the nitro group followed by treatment of the resulting diamine with trimethyl orthoformate and piridinium *p*-toluensulfonate, and the Bischler-Napieralski reaction furnished (+)-trigonoliimine in 7.5% overall yield and 84% *ee*. It is noteworthy that, employing a quinidine-derived bifunctional catalyst and following exactly the same procedure, (–)-trigonoliimine A could be obtained in 6.8% overall yield and 73% *ee* [54].

**Scheme 23:**
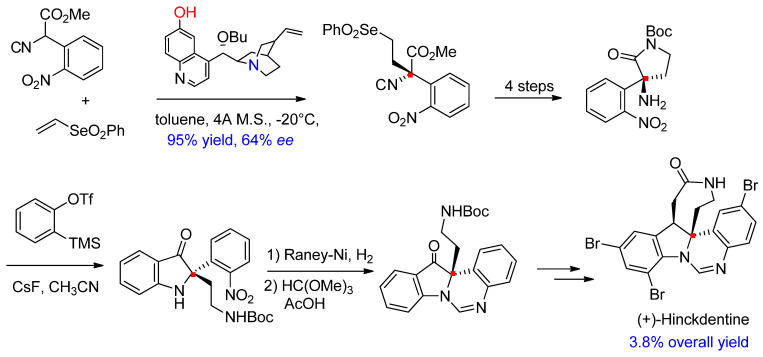
Catalytic asymmetric Michael addition of methyl α-(2-nitrophenyl)-α-isocyanoacetate to vinyl phenyl selenone as key step for the total synthesis of (+)-Hinckdentine A.

The catalytic asymmetric Michael addition of methyl α-(2-nitrophenyl)-α-isocyano acetate to phenyl vinyl selenone in the presence of a quinidine-derived bifunctional catalyst was the key step for the enantioselective construction of the (+)-Hinckdentine A (Scheme 23). This is a marine alkaloid isolated from the bryozoan *Hincksinoflustra denticulata* collected from Tasmania’s eastern coast [55].

**Scheme 24:**
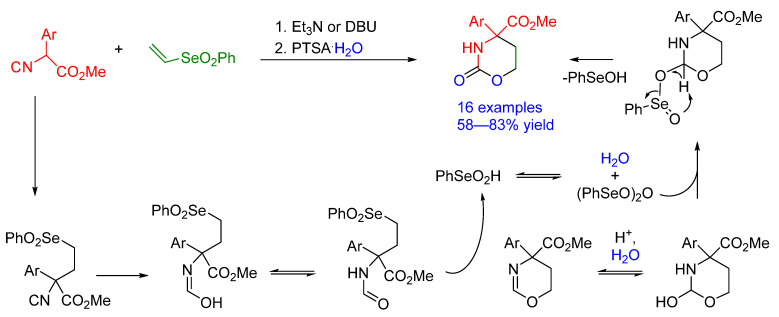
Synthesis of 1,3-oxazinan-2-ones.

In 2014, Zhu reported the reactions of 2-substituted isocyanoacetates, alkenyl selenones and water to afford 4,4-disubstituted 1,3-oxazinan-2-ones in good to excellent yields [56]. In this process four new bonds are formed. 1,3-Oxazinan-2-one ring is a privileged scaffold found in bioactive and natural compounds displaying antibacterial, anti-inflammatory, anti-diabetes, and anti-HIV activities. The process was carried out in two steps by treatment with a catalytic amount of a base followed by the addition of hydrated *p*-toluenesulfonic acid (PTSA^.^H_2_O). Based on the results of control experiments carried out with ^18^O labeled water, the following reaction steps were suggested: (a) the Michael addition, (b) the partial hydrolysis of the isocyanide which generates the formamide, (c) the nucleophilic displacement of the phenylselenonyl group by the amide oxygen with formation of a 5,6-dihydro-4*H*-1,3-oxazine and release of benzenseleninic acid (in equilibrium with the benzeneseleninic anhydride, BSA), (d) a second addition of water under acidic condition to generate a 1,3-oxazinan-2-ol, (e) the formation of the seleninate by reaction with BSA and finally, (f) the oxidation to 1,3-oxazinan-2-one with elimination of benzeneselenenic acid (Scheme 24).

**Scheme 25:**
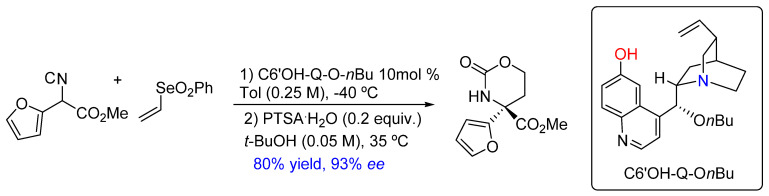
One-pot enantioselective synthesis of a 1,3-oxazinone.

Thus, the phenyl selenonyl group acts consecutively as an alkene activator, a leaving group and a latent oxidant. The asymmetric variant of this reaction employed a quinine-derived bifunctional organocatalyst to generate the 4,4-disubstituted 1,3-oxazinan-2-one with an excellent enantiocontrol (Scheme 25).

**Scheme 26:**
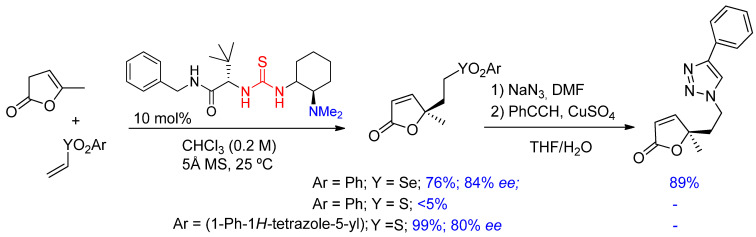
Enantioselective vinylogous addition of deconjugated substituted butenolides to vinyl chalcogenones.

In 2016, Simlandy and Mukherjee developed the enantioselective vinylogous addition of deconjugated substituted butenolides to vinyl selenones [57]. The reaction was catalyzed by a thiourea derivative and gave access to γ, γ-disubstituted butenolides as the Michael adducts in good yield and enantioselectivity starting from both alkyl or aryl substituted compounds. Interestingly, the vinyl phenyl sulfone resulted completely unreactive (Scheme 26) under the condition employed. On the contrary, the vinyl (1-phenyl-1*H*-tetrazol-5-yl)sulfone gave the adduct in comparable yield and enantioselectivity with the selenium reagent. As reported in the same scheme, the selenone adduct was practically converted into an azide and finally into a triazole by Cu-catalyzed click-reaction with phenylacetylene.

**Scheme 27:**
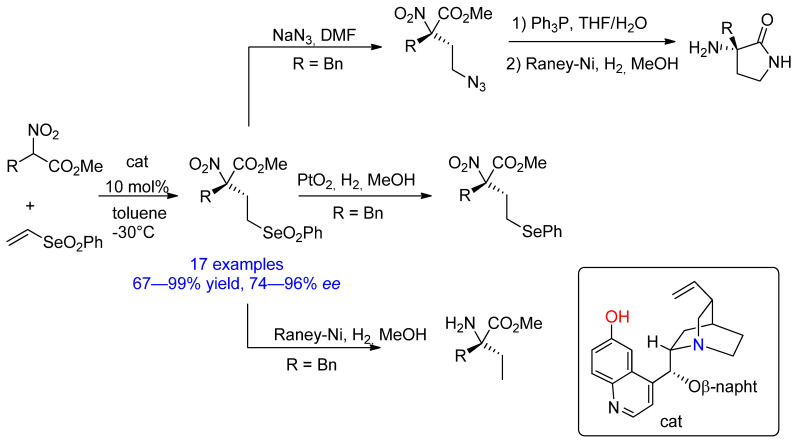
Enantioselective addition of 2-alkyl-2-nitroacetates to phenyl vinyl selenones and chemical transformations.

In 2019, Zhu and coworkers developed a highly enatioselective conjugate additionbetween 2-alkyl-2-nitroacetates and phenyl vinyl selenones in the presence of a 6′-OH quinine derived bifunctional catalyst [58]. The Michael adducts could be further transformed into quaternary α-aminoacids or other densely functionalized compounds due to the chemical versatility of the nitro and phenyselenonyl functionalities. The adducts were obtained in 67–99% yield and 74–96% *ee*. In another experiment, the phenyselenonyl group was reduced into a selenide retaining the unmodified nitro group with an excellent level of chemoselectivity. Alternatively, this group was reductively removed with the simultaneous reduction of the nitro group. These and other transformations are reported in Scheme 27.

**Scheme 28:**
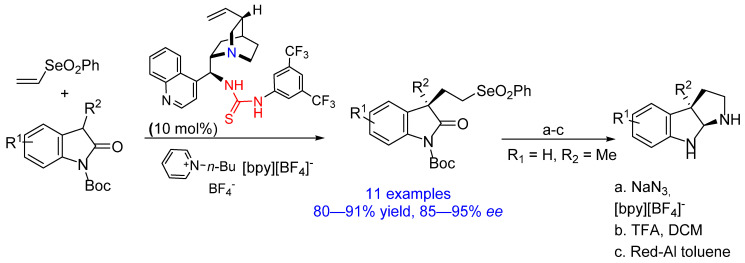
Enantioselective organocatalyzed Michael additions in ionic liquid and application to the synthesis of a pyrroloindoline.

Finally, hydrogen-bond-mediated catalytic processes with vinyl selenone in ionic liquids were reported [59]. Variously substituted 2-oxindoles were added to phenyl vinyl selenone at room temperature in the presence of a *Cinchona*-derived thiourea catalyst in a pyridine-based ionic liquid with good yields and an excellent enantiocontrol (Scheme 28).

Easy transformations of an enantioenriched Michael adduct furnished the corresponding pyrroleindoline without loss of enantiomeric purity. This heterocyclic scaffold is typical of (−)-physostigmine and other biologically active compounds.

## 5. Cycloaddition Reactions of Vinyl Selenones

**Scheme 29:**
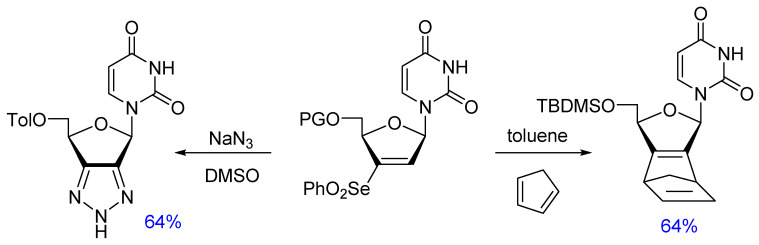
Early examples of cycloadditions with vinyl selenones.

As reported from previous discussion, vinyl selenones have been well investigated as Michael acceptors, but few methods refer to their use as 2π partners in cycloaddition reactions. Seminal examples were described by Chattopadhyaya et al. in 1990 [39] using a 5′-O-protected vinyl selenone-modified nucleoside and sodium azide at 20 °C for 5 h (Scheme 29). A triazole was recovered with an acceptable yield (64%) through a [3+2]-cycloaddition followed by elimination of benzenseleninc acid. In the same paper authors also explored the Dies-Alder reaction with cyclopentadiene. After four days at 60 °C, a product was obtained in 64% yield.

**Scheme 30:**
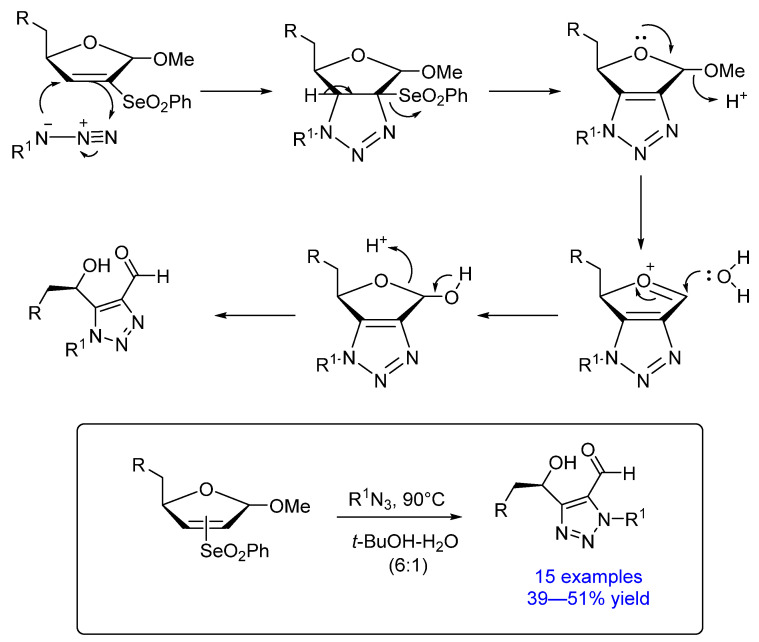
Vinyl selenones-modified carbohydrates for the synthesis of 1,2,3-triazoles.

More recently, in 2014, Pathak and co-workers described the synthesis of enantiomerically pure 1,4,5-trisubstituted-1,2,3-triazoles starting from four different vinyl selenones derived from *d*-xylose and *d*-glucose [60]. The cascade transformation is initiated by a regioselective1,3-dipolar cycloaddition with organic azides followed by elimination of benzenseleninic acid. The method did not require any catalyst or metal. The opening of the sugar ring, due to the cleavage of the acetal bond, afforded the trisubstituted triazoles (Scheme 30).

**Scheme 31:**
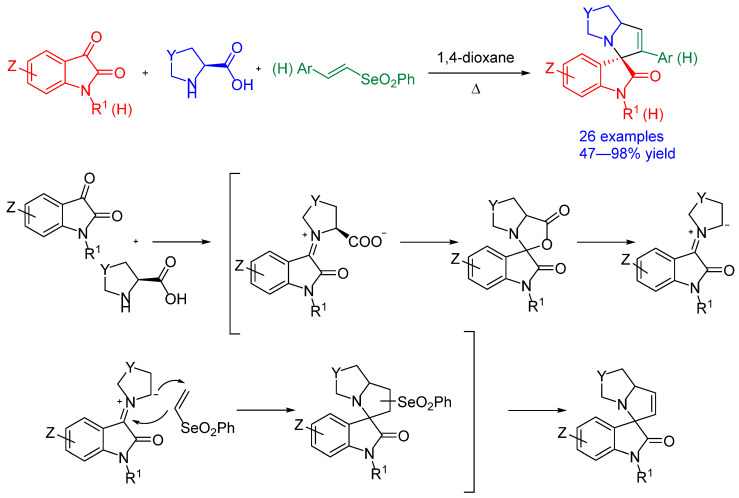
Three component [3+2] cycloaddition/elimination reaction for the synthesis of spiro oxindoles.

In 2021 a multicomponent [3+2] cycloaddition/elimination cascade for the synthesis of spirooxindole pyrrolizines has been developed [61]. The reaction tolerates the presence of a range of functional groups. Reactions were performed in 1,4-dioxane at reflux with in situ generated azomethine ylides and vinyl selenones as dipolarophiles (Scheme 31).

The 1,3-dipole was obtained by decarboxylative condensation of isatins and a secondary α-aminoacids. Products were obtained in good to excellent yields and high diasteroselectivity, regardless of the nature and position of the substituent on the isatin derivative. *L*-proline gave better yields in respect to other secondary aminoacids such as the *trans*-4-hydroxy-*L*-proline, *L*-thioproline, and sarcosine. Excellent regioselectivity were observed with aryl-substituted selenones. By analogy with other dipolarophiles, the three-component cycloaddition can proceed through the following steps: (a) the condensation between the isatin and the α-aminoacid to generate an iminium ion in equilibrium with a cyclic intermediate, (b) the decarboxylation for the formation of the azomethine ylide, (c) the [3+2]-cycloaddition, (d) the spontaneous loss of benzenseleninic acid to generate a double bond.

The elimination step is not common with other dipolarophiles. Experiments with other chalcogen-containing functional groups, such as selenoxide, sulfoxide, and sulfones, were unsuccessful.

## 6. Transition-Metal Catalyzed Cross-Coupling of Vinyl Selenones

**Scheme 32:**
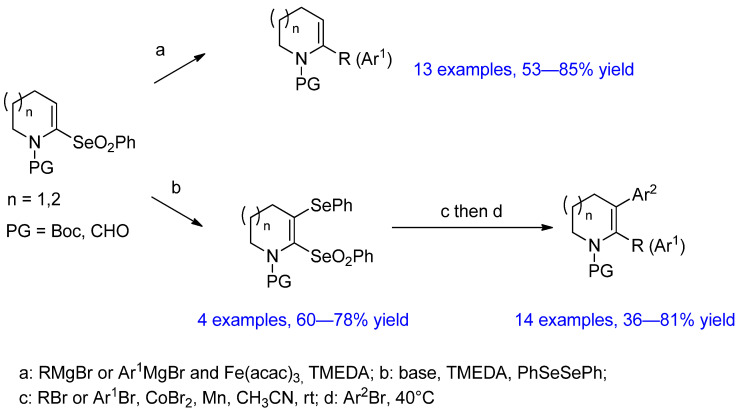
α- and β functionalization of piperidine or azepane ene-formamides or carbamates.

The investigation of vinyl selenones as electrophiles in transition-metal catalyzed cross-coupling reactions has been explored by Beng in 2015. Scheme 32 shows the iron catalyzed α-carbofunctionalization of piperidine and azepane ene-formamides or carbamates with alkenyl, aryl, heteroaryl, and allyl Grignard reagents (route a).

The electron-withdrawing nature of the selenonyl group also facilitated the β-functionalization of the aza-heterocycles by lithiation and trapping with selenium electrophiles (route b). The aza-heterocycles were then vicinally difunctionalized with different carbon partners (route c–d), taking advantage of the diverse reactivity of the two selenylated functional groups [62]. In fact, the coupling between the β-selenyl group and the aryl bromide required warming to 40 °C.

## 7. Conclusions

**Scheme 33:**
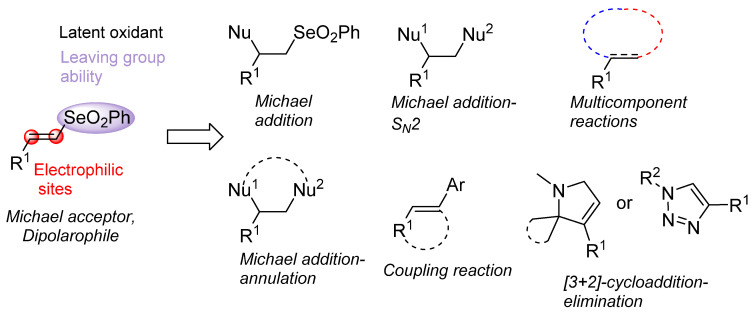
Vinyl selenones as reactive species in organic synthesis.

In this review, we discussed recent developments in vinyl selenone chemistry. The reactivity of vinyl selenones has been explored in a variety of Michael-initiated multiple bond-forming reactions, cycloadditions and coupling reactions. The simple preparation, the ease of handling, and the variety of possible transformations make the vinylselenones interesting building blocks with useful applications in (hetero)cycle synthesis, total synthesis of natural products and modular construction of drug-like molecules. Organocatalyzed protocols with cinchona derivatives or other privileged organocatalysts gave access to densely functionalized compounds with a high level of enantiocontrol. We hope that the chemistry described in this review can stimulate further investigations in the underexplored field of selenone-mediated transformations (Scheme 33).
